# Genetic Diversity of *Pinus nigra* Arn. Populations in Southern Spain and Northern Morocco Revealed By Inter-Simple Sequence Repeat Profiles †

**DOI:** 10.3390/ijms13055645

**Published:** 2012-05-10

**Authors:** Angela Rubio-Moraga, David Candel-Perez, Manuel E. Lucas-Borja, Pedro A. Tiscar, Benjamin Viñegla, Juan C. Linares, Lourdes Gómez-Gómez, Oussama Ahrazem

**Affiliations:** 1 Department of Agricultural Technology and Science and Genetics, Faculty of Pharmacy, Institute of Botany, University of Castilla-La Mancha, Campus Universitario s/n, Albacete, E-02071, Spain; E-Mails: Angela.rubio@uclm.es (A.R.-M.); Marialourdes.gomez@uclm.es (L.G.-G.); 2 Department of Agricultural Technology and Science and Genetics. ETSIA, University of Castilla-La Mancha, Campus Universitario s/n, Albacete, E-02071, Spain; E-Mails: David.Candel@uclm.es (D.C.-P.); ManuelEsteban.Lucas@uclm.es (M.E.L.-B.); 3 Training Center and Experimental Forestry, Cazorla, E-23470, Spain; E-Mail: pedroa.tiscar@juntadeandalucia.es; 4 Faculty of Experimental Sciences, University of Jaén (B3-158), Campus Las Lagunillas, s/n E-23009, Jaén, Spain; E-Mail: bvinegla@ujaen.es; 5 Department of Physical Systems, Chemical and Natural Sciences, Pablo de Olavide University, Ctra. Utrera km. 1, Sevilla, E-41002, Spain; E-Mail: jclincal@upo.es; 6 Albacete Science and Technology Park, Campus Universitario s/n, Albacete, E-02071, Spain

**Keywords:** *Pinus nigra*, genetic diversity, populations, ISSR

## Abstract

Eight *Pinus nigra* Arn. populations from Southern Spain and Northern Morocco were examined using inter-simple sequence repeat markers to characterize the genetic variability amongst populations. Pair-wise population genetic distance ranged from 0.031 to 0.283, with a mean of 0.150 between populations. The highest inter-population average distance was between PaCU from Cuenca and YeCA from Cazorla, while the lowest distance was between TaMO from Morocco and MA Sierra Mágina populations. Analysis of molecular variance (AMOVA) and Nei’s genetic diversity analyses revealed higher genetic variation within the same population than among different populations. Genetic differentiation (*Gst*) was 0.233. Cuenca showed the highest Nei’s genetic diversity followed by the Moroccan region, Sierra Mágina, and Cazorla region. However, clustering of populations was not in accordance with their geographical locations. Principal component analysis showed the presence of two major groups—Group 1 contained all populations from Cuenca while Group 2 contained populations from Cazorla, Sierra Mágina and Morocco—while Bayesian analysis revealed the presence of three clusters. The low genetic diversity observed in PaCU and YeCA is probably a consequence of inappropriate management since no estimation of genetic variability was performed before the silvicultural treatments. Data indicates that the inter-simple sequence repeat (ISSR) method is sufficiently informative and powerful to assess genetic variability among populations of *P. nigra*.

## 1. Introduction

European black pine (*Pinus nigra* Arn.) is a tertiary relictual species belonging to the group of Mediterranean pines [1]. Their forests are included in the EU endangered natural habitat listing requiring specific conservation measures (Resolution 4/1996 by the Convention on the Conservation of European Wildlife and Natural Habitats) due in part to a lack of basic understanding of their regeneration biology [2–4]. *P. nigra* is one of the oldest European pine species, descending from a group that already existed in the Cretaceous (100 million years ago) [5]. *P. nigra* is a widespread species, with a discontinuous range that extends from North Africa through the northern Mediterranean and eastwards to the Black Sea. It is also found on the islands of Corsica and Sicily. The fragmented distribution has led to morphological variations, which are difficult to interpret and have resulted in several diverse classifications. Relatively little is known about its long history, although in the Tertiary it was more widespread than today and it seems to have shifted over time from coastal areas to its current mountain locations, since the dry cold climate of these areas resembles that of bygone glacial periods [6]. Migratory movements, which occurred during interglacial warm periods, brought the emergence of various hybrid populations that later became genetically isolated, thus contributing to its difficult taxonomic characterization [7].

The species *P. nigra* is divided into five subspecies: *P. nigra* ssp. *nigra* from the Alps; *P. nigra* ssp. *laricio* from Corsica and Sicily; *P. nigra* ssp. *pallasiana* from Turkey and Crimea; and both *P. nigra* ssp. *salzmannii* and *P. nigra* ssp. *mauretanica* from North Africa. In Spain, the predominant subspecies is ssp. *salzmannii*, with two varieties that are also identified within the subspecies *P. nigra* ssp. *Salzmannii*: the *hispanica* variety located in the Iberian Central System and Betic Cordilleras and the *pyrenaica* variety, which extends throughout Castellón, Aragón and Catalonia [7].

Trees are more vulnerable than annual plants to rapid climate changes since they are not able to respond by migration or genetic selection within a short period of time. High genetic variability of forest species is therefore important for the processes of adaptation to biotic and abiotic stresses in order to ensure the viability of the species. Since the pressure on natural resources has brought about significant losses in diversity, especially in tree species, it is essential to gain a comprehensive idea of genetic variability amongst populations in order to provide a basis for *in situ* conservation, exploitation of genetic resources and forest management [8–11]. In recent years, a wide array of DNA markers has been used in genetic studies on forest populations to assess neutral variation and to analyze population structure, gene flow, phylogenetic relationships and genetic linkage. Inter-simple sequence repeat (ISSR) regions of the nuclear DNA, which are dominant molecular markers with high reproducibility, can be used to detect DNA variability at different levels, from single base changes to deletions and insertions [12–16]. Furthermore, polymorphisms can be detected without any previous knowledge of a tree’s DNA sequence.

Numerous studies have researched the genetic variation in natural populations of *P. nigra* using DNA markers [6,7,17]. These studies reveal that most diversity is typically partitioned within populations, with a low genetic differentiation among populations. The primary objective of this study was to assess genetic variation within and among populations of *P. nigra* from seven populations in Southern Spain and one population in Northern Morocco, in order to develop the genetic background for their conservation and restoration.

## 2. Results and Discussion

Two *Pinus nigra* samples were selected for preliminary experiments to determine optimal amplification reaction conditions and primer screening for ISSR. 12 primers did not result in any amplification, 15 gave unclear profiles in some samples and only eight out of the 35 primers tested resulted in well separated bands. On the basis of this data, the 160 samples included in the study were analyzed with eight primers (Table 1). Most loci were polymorphic within each population with respect to presence and absence of bands. Examination of intra-population genetic diversity revealed the highest values of Nei’s genetic diversity (0.242), Shannon information index (0.366) and polymorphic loci (79.17%) among samples from ArCU populations and the lowest values of Nei’s genetic diversity (0.123), Shannon information index (0.178) and polymorphic loci (29.17%) among samples from PaCU populations (Table 2).

Total genetic diversity for *P. nigra* in this study (*H_E_* = 0.175) was lower than other *Pinus* species similarly researched with ISSR markers, such as *Pinus tabuleaformis* (0.4152, [18]), *Pinus koraiensis* (0.3477, [19]), *Pinus sibirica* (0.2699, [20]), *Pinus sylvestris* (0.2620, [21]; 0.217–0.310, [22]), and higher than *Pinus squamata* (0.029, [23]). The differences in the levels of genetic diversity among these species may be related to geographic distribution, number of population tested, population size of the species and the effect of climate changes during the last glacial maximum.

The populations from the Cuenca region showed the highest Nei’s genetic diversity except the population PaCU. TrCU and ArCU are natural populations and have been neither exploited nor managed.

The low diversity of PaCU and YeCA can be explained by the forest management practices applied to this population. Since the end of the 19th century, the Spanish black pine forest stand at PaCU and YeCA has been managed under the shelterwood system with a 100–120-year rotation and 20–30-year regeneration period. This Spanish black pine stand was divided into compartments up to 111 hectares in surface, delineated by roads, streams, rocky outcrops and other spatial features. Individual compartments or a number of aggregated ones were established as management units, and for each management unit tactical planning considerations, *i.e.*, where and when to apply silvicultural treatments, were defined. According to the shelterwood method context, some seedling trees (from 1 to 4 per ha) were left standing. Thus, for each regeneration period, the new trees were seeded naturally with seeds from the seedling trees. This silvicultural system has continued in Cuenca and Cazorla for forest stand regeneration, leading to a decrease in genetic diversity in these populations since before the silvicultural treatments no estimation of genetic variation of PaCU and YeCA has been projected to provide an appropriate strategy for sampling and propagation.

In 1893, NaCA was first managed under the shelterwood method with a shelter-phase of 20 years and a rotation of 120 years. In 1920 an uneven-aged system was applied and in 1944, an ideal reverse-J diameter distribution was achieved to resolve the unsuccessful natural regeneration. This silvicultural treatment may explain the lower Nei’s genetic diversity in comparison with the two natural populations of *P. nigra* from Cuenca’s region (TrCU and ArCU). Tiscar-oliver *et al.* [24] studied the impact of the management in PaCU and NaCA in the structure and composition of *P. nigra* populations. The authors concluded that the number of large trees and the diameter classes decrease in both populations over time; however the uneven-aged silvicultural method showed higher values of both variables. No management data were recorded for TaMO, MA and PaCA.

Pair-wise Nei’s distances [25] were calculated for all the populations. The highest inter-population average distance (0.283) was between PaCU and YeCA, while the lowest distance (0.031) was between TaMO and MA populations (Table 3).

The low distance between TaMO and MA is logical since Southern Spain and Northern Morocco have the same geological origin. Several authors have highlighted the floristic continuity of both sides of the Strait of Gibraltar and have thus proposed the creation of a Tingitanian-Aljibean floristic sector [26]. Exchange of terrestrial organisms between Africa and Europe at the western margin of the Mediterranean Sea is well documented. During the Betic crisis 16–14 Myr, marine transgressions through the Betic Strait separated the Betic-Rif mountain belt [27] from the Iberian mainland. Dispersal throughout the Betic arch, which consisted of a chain of small mountain ridges, was possible. Finally, promoted by the further orogenic uplift of the Alboran basin between Iberia and Africa, the southernmost part of the insular Betic region connected to the African continent and formed the present-day Rif Mountains. In the Pliocene, the tectonic movement of the African plate towards the European plate and again an uplift of the Alboran region closed the Strait of Gibraltar c. 5.59 Myr [28,29]. The resulting land bridge between Africa and Europe persisted for a long period of time. As a consequence, the Mediterranean Sea steadily desiccated (Messinian salinity crisis; [30]) and dispersal was possible not only across the land bridge but also throughout large parts of the Mediterranean basin. At 5.33 Myr, the Strait of Gibraltar reopened and again separated Africa from Europe. As of present, Southern Spain and Northern Morocco share approximately 75% of their flora.

**Figure 1:**
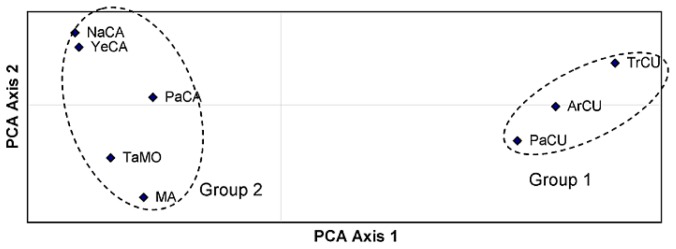
Principal component analysis (PCA) plot of the eight populations based on the first two principal axes (first axis = 51.63% and the second = 22.16%). Population names are abbreviated as in Table 4.

The average distance between populations from the Cuenca region (0.064) was lower than the populations from Cazorla (0.120) suggesting different patterns of genetic exchange and genetic divergence between the two regions. Based on these distances, principal component analysis (PCA) analysis was computed showing the presence of two major groups (Figure 1). Group 1 contains all populations from Cuenca while Group 2 contains populations from Cazorla, Sierra Mágina and Morocco, thus indicating cross-migration from one region to another.

The genetic differences between the Cazorla and Cuenca regions may be due to several factors such as natural barriers, agricultural and forest harvesting, local adaptation and management methods [24].

**Figure 2:**
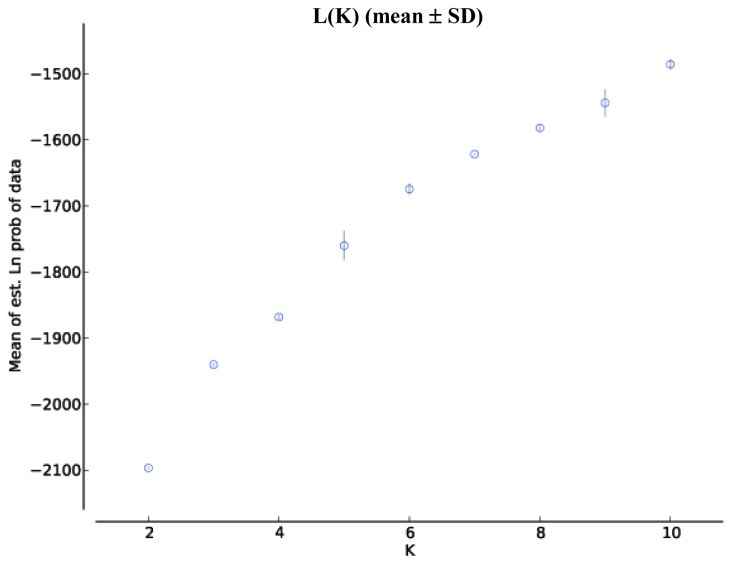
The relationship between the log probability of the data and the number of clusters K using the ISSR data.

**Figure 3:**
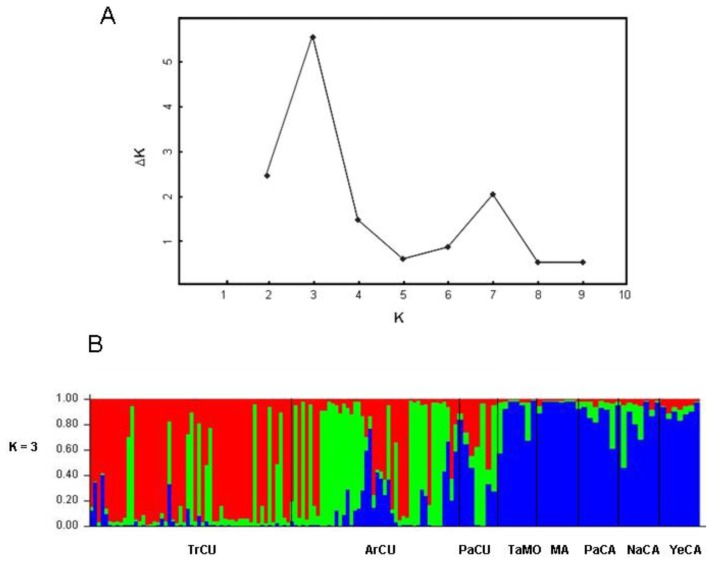
STRUCTURE analysis of eight populations of *Pinus nigra* sampled to assess inter-simple sequence repeat markers. (**A**) K = 3 appeared to be the optimal number of clusters by showing the ΔK at its peak; (**B**) Results based on K = 3 using a Bayesian framework implemented in the STRUCTURE program across individuals from the studied populations.

The population structure of *P. nigra* inferred using the method of Pritchard *et al.* [31], was carried out by STRUCTURE (version 2.3; University of Chicago: Chicago, IL, USA, 2004). The plot of the average log likelihood values over 10 runs for K values ranging from 2 to 10 shows that the log likelihood estimates increase progressively as K increases (Figure 2). The optimal value of K was 3 as determined by the ΔK statistic STRUCTURE (Figure 3). We calculated the proportions of membership (q1 for cluster 1, q2 for cluster 2, and q3 for cluster 3, with q1 + q2 + q3 = 1) of each region Cazorla, Cuenca, Sierra Mágina and Morocco. The populations from Cazorla, Morocco and Sierra Mágina were assigned to cluster 3, with a high percentage membership q3 = 0.85 for Cazorla, q3 = 0.89 for Morocco and q3 = 0.93 for Sierra Mágina which corresponds to Group 2 in the PCA analysis; all the populations from Cuenca region were assigned to cluster 1 and 2 with percentage membership of q1 = 0.35 and q2 = 0.54.

Based on the AMOVA analysis, only 9% of the total variation resides among the *P. nigra* population, while 52% is attributed to the differences among individuals within populations and 39% among regions. The contributions from each of the three sources were significantly greater than 0, indicating statistically significant genetic differentiations among and within populations (*P* < 0.01 in the AMOVA tests). A relatively high level of differentiation among populations in the studied regions was found. Our data showed that 9% of the total genetic variation was attributed among populations which were much higher than the one reported by Naydenov *et al.* [6] analyzing populations of *P. nigra* from Bulgaria. The most genetic variation in *P. nigra* is within populations rather than between them, indicating relatively restricted population differentiation. Such a pattern of population genetic structure has been found in all *P. nigra* populations in different regions [6,7,17]. The high level of observed genetic diversity within populations is in accordance with the findings of Hamrick and Godt [32] who suggested that gymnosperms, with long lifespans, high outcrossing rates and fecundity, maintain high intra-population genetic diversity. The majority of conifer populations studied display relatively high levels of genetic diversity and low levels of interpopulational differentiation compared to other groups of plants. Generally speaking they also display genotypic frequencies consistent with Hardy-Weinberg equilibrium or an excess of heterozygotes. The extraordinary high differentiation among regions is probably due to the action of genetic drift and relatively low levels of gene flow. The same pattern was reported in other studies within conifer species [33,34].

**Figure 4:**
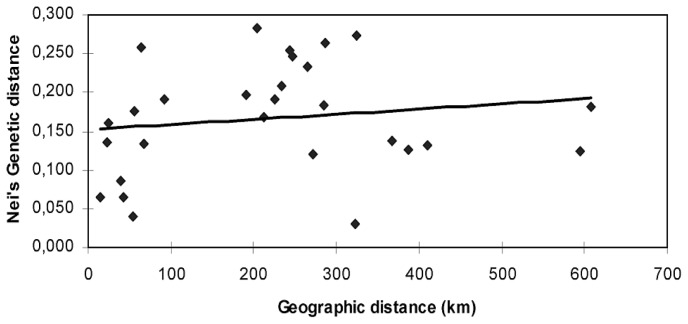
Relationship between genetic and geographic distances among the populations of *Pinus nigra* included in this study. The line is the regression fitted to the data. *X*-axis geographic distance in kilometers; *Y*-axis Nei’s genetic distance.

Both a small positive correlation of genetic diversity with a mean annual precipitation (*r* = 0.3) and a medium negative correlation with a mean temperature (*r* = −0.4) were found. Geographic distances between population-pairs were not correlated with the corresponding genetic distance (Mantel test: *r =* 0.151, *P =* 0.196; Figure 4), revealing different patterns of genetic diversity among populations of *P. nigra*, irrespective of their geographical distribution. These findings also support the clustering of the population from Morocco (TaMO) with the population from Sierra Mágina (MA). The climate changes during the late Pliocene and the Pleistocene in the Mediterranean region are highly complex and palaeoenvironmental data have demonstrated century-to-millennial climate variability, with rapid changes [35,36]. A recent study conducted by Medial and Diadema [37] who identified 52 refugia in the Mediterranean bioclimatic region and confirmed the role played by the three major peninsulas, with a shared total of 25 refugia. The locations of the phylogeographically defined refugia are significantly associated with the 10 regional hotspots of plant biodiversity, with 26 of these refugia (*i.e.*, 50%) occurring within the hotspots. Hotspot 2, which encompasses the Baetic–Rifan complex, supports the relatedness of the Morocco population with those of southern Spain.

The data reported in this study should be validated using a large size of samples from PaCU, TaMO, MA, PaCA, NaCA and YeCA populations and should be complemented using codominant markers.

## 3. Experimental Section

### 3.1. Plant Material and Population Selection

**Figure 5:**
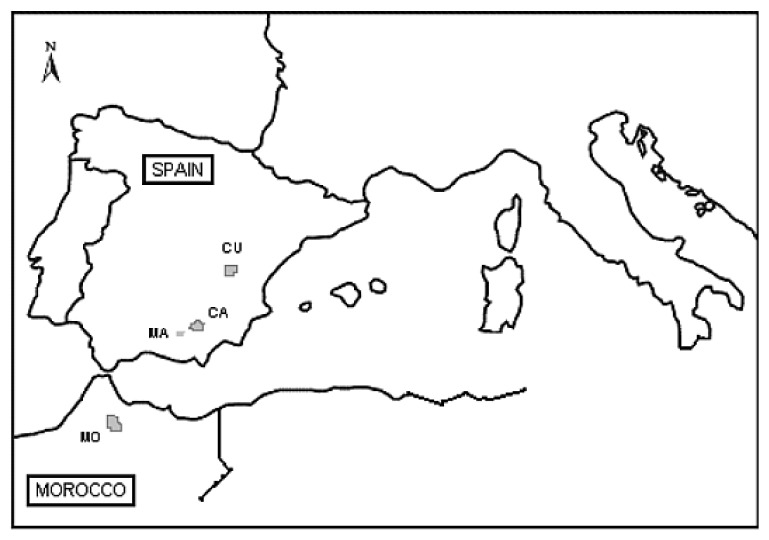
Geographic locations of populations sampled from Spain and Morocco. CU (Cuenca Mountains); CA (Cazorla Mountains); MA (Sierra Mágina); MO (Atlas Range).

One hundred sixty samples of *Pinus nigra*, collected in October 2010 from eight populations, were sampled from four regions: 1-Cuenca (Spain), 2-Talassemtane (Morocco), 3-Sierra Mágina (Spain) and 4-Cazorla (Spain) (Table 4 and Figure 5). All the areas studied fall within a Mediterranean type climate, with snowfalls and frost common during the winter and hot, dry summers. Rock parent material is Cretaceous limestone at all sites, and soils are calcium rich and mainly shallow.

### 3.2. DNA Extraction

DNA was extracted from 150 to 300 mg of needle/bud material using a modified method [38]. Leaf material was then ground to a fine powder in liquid nitrogen and placed in a microcentrifuge tube with 2 mL of extraction buffer (2% CTAB, 100 mM Tris-HCl pH 8.0, 20 mM EDTA, 1.4 M NaCl, and 0.01% proteinase K) plus 40 μL of 2-mercaptoethanol. Following incubation at 65 °C for 30 min, 1.4 mL of chloroform:isoamyl alcohol (24:1) was added, mixed and centrifuged at 8000 rpm for 30 min; the supernatant was transferred to a new tube and then the process was repeated three times. DNA was precipitated with isopropanol (2/3 volume of supernatant), then centrifuged at 8000 rpm for 30 min, the supernatant discarded and the pellet washed in 70% ethanol containing 10 mM ammonium acetate for 20 min. The pellet was dissolved in 100 μL of TE buffer (10mM Tris-HCl pH 7.4, 1 mM EDTA) and the DNA was reprecipitated with ½ volume of ammonium acetate 3 M and 2.5 volumes of ethanol. After centrifuging at 8000 rpm for 30 min, the pellet was redissolved in TE buffer with 10 μg/mL RNase and incubated at 30 °C for 30 min. The extracted DNA was quantified with a spectrophotometer, diluted to 30 ng/μL in TE and then stored at −20 °C for further analysis.

### 3.3. DNA Amplification

15 and 30 ng of genomic DNA were amplified in a volume of 25 μL containing 10 mM Tris-HCl, pH 9.0, 1.5 mM MgCl_2_, 200 μM each of dATP, dCTP, dGTP, and dTTP, 0.4 μM primer, and 1 unit of Taq DNA polymerase using a thermal cycler (MJ-Mini, BioRad). The cycling program began with an initial 2 min at 94 °C followed by 40 cycles at 94 °C for 45 s, 52–62 °C for 45 s and 72 °C for 2 min plus a final 10 min at 72 °C and storage at 4 °C. Water was the negative control while the positive control showed an amplicon of expected size = 320 bp. The sequences of primers are shown in Table 1. Amplification products were separated by electrophoresis in 2% agarose gel containing 1 μg/mL ethidium bromide and TAE buffer. Ten microlitres of amplified DNA were mixed with 3 μL sample buffer (1.2 mg/mL; 125 mg/mL Ficoll) and 10 μL was applied in each well of the gel. DNA molecular weight markers (1 kb, Promega) were then added to each gel. The gels were run at a current of 50 mA until the bromophenol had migrated 10 cm from the well. The bands were then visualized under UV light and photographed. To ensure the reproducibility of the method, the procedure was repeated three times for each concentration of genomic DNA and primer.

### 3.4. Data Analysis

We analyzed ISSR data based on both allele and phonotypic frequencies. Polymorphic bands were selected at the 95% level (two-tailed test) for use in further analyses. Data matrices were analyzed using POPGENE version 1.32 [39] with the assumption that the populations were in Hardy–Weinberg equilibrium. The following parameters were determined: percentage of polymorphic loci (*PPL*), number of alleles per locus (*n_a_*), effective number of alleles per locus (*n_e_*), genetic diversity (*H_E_* = expected heterozygocity), Shannon’s index diversity (*I*) and genetic differentiation among populations (*G_ST_*).

We examined the hierarchical genetic variation across the geographic range of the populations studied using an analysis of molecular variance (AMOVA) determined with GenAlEx 6.41 [40]. To test the correlations among genetic distances and geographic distances of populations, Mantel’s [41] tests were conducted. The randomized Mantel statistic (Pearson correlation, *r*) was calculated and assessed by permuting the arrangement of distance matrices. The *P* value associated to the observed correlation is the proportion of such permutations that lead to a higher correlation coefficient than the one observed. The Pearson correlation coefficients between pairs of genetic diversity and climate factor (temperature and precipitation) for these populations were analyzed with Excel (Microsoft Office Excel 2003, Microsoft Corporation: Redmond, WA, USA, 2003). We tested the null hypothesis that genetic distance and geographical distance matrices are not correlated against the alternative hypothesis that the two matrices are correlated using GenAlEx 6.41. Genetic relationships among populations were studied via principal component analysis (PCA) using GenAlEx 6.41 [40].

Bayesian assignment tests were applied to estimate the number of genetic clusters and to evaluate the degree of admixture among them using Structure v2.3.3 [42]. Structure was run with a “burn-in” setting of 100,000 followed by 20,000 MCMC iterations using the admixture model with sampling localities as prior population assignment and with allelic frequencies correlated among populations. Ten runs were performed for each value for *K* ranging from 2 to 10. The most likely value for *K* was calculated with Structure Harvester [43] using the statistic *ΔK*, which represents the greatest rate of change between each subsequent *K* value [44].

## 4. Conclusions

Genetic differentiation among populations of *Pinus nigra* in the studied regions is essential for conservation of genetic resources. If genetic diversity is to be preserved an estimation of genetic variability of populations should be achieved before initiating a planting strategy. The low genetic diversity observed in PaCU and YeCA is probably a direct consequence of inappropriate management since no estimation of genetic variability was carried out before the silvicultural treatments. We strongly recommended testing the genetic variability of populations before any management. Our results indicate that ISSR are sufficiently informative for assessing genetic variability. Because of the dominant nature of the ISSR markers, the application of codominant molecular markers should be undertaken to complement the data reported here.

## Figures and Tables

**Table 1:** Inter-simple sequence repeat (ISSR) primers and their sequences used in the present study.

Primer Name	Sequence (5′-3′)	Tm (°C)
ISCS14	AGTGAGTGAGTGAGTGAGTGA	52
ISCS17	DBDBCACCACCACCACCAC	62
ISCS19	HVHGTGGTGGTGGTGGTG	62
ISCS20	DHBCGACGACGACGACGA	62
ISCS21	BDBACAACAACAACAACA	52
ISCS34	TGTGTGTGTGTGTGTGRC	52
ISCS41	CTCCTCCTCCTCCTCCTC	62
ISCS69	CACACACACACACACAA	52

B: G + T + C; D: G + A + T; H: A + C + T; V: G + C + A.

**Table 2:** Genetic diversity of *Pinus nigra* populations in this study.

Population	PPL (%)	*n_a_*	*n_e_*	*H_E_*	*I*
TrCU	70.83	1.542	1.372	0.218	0.331
ArCU	79.17	1.625	1.410	0.242	0.366
PaCU	29.17	0.875	1.222	0.123	0.178
TaMO	54.17	1.208	1.368	0.207	0.303
MA	62.50	1.375	1.273	0.170	0.268
PaCA	29.17	0.875	1.239	0.130	0.185
NaCA	45.83	1.167	1.326	0.180	0.262
YeCA	37.50	1.083	1.228	0.133	0.199
Mean	51.04	1.219	1.305	0.175	0.262

PPL: percentage of polymorphic loci; *n_a_*: number of Different Alleles; *n_e_*: number of Effective Alleles; *H_E_*: expected Heterozygosity; *I*: Shannon’s Index diversity.

**Table 3:** Inter-population genetic distances calculated by Nei’s method. Above the diagonal are values of Nei’s unbiased genetic distances, those below the diagonal are Nei’s genetic distances. The underlined values are maximum or minimum genetic distances.

	TrCU	ArCU	PaCU	TaMO	MA	PaCA	NaCA	YeCA
**TrCU**	-	0.037	0.079	0.249	0.267	0.223	0.252	0.244
**ArCU**	0.040	-	0.057	0.115	0.113	0.158	0.196	0.187
**PaCU**	0.087	0.065	-	0.168	0.171	0.176	0.230	0.268
**TaMO**	0.258	0.124	0.181	-	0.019	0.111	0.121	0.116
**MA**	0.274	0.120	0.183	0.031	-	0.121	0.161	0.177
**PaCA**	0.233	0.168	0.191	0.126	0.134	-	0.118	0.143
**NaCA**	0.264	0.208	0.247	0.138	0.176	0.136	-	0.046
**YeCA**	0.254	0.198	0.283	0.131	0.191	0.160	0.064	-

**Table 4:** Sampling of accessions from different populations of *P. nigra* used in this study.

Country, Locality	Site	Population Code	Elev. (m)	Longitude	Latitude	*n*	Mean Annual Temperature (°C) a	Mean Annual Precipitation (mm) a
Spain, Cuenca (CU)	Tragacete	TrCU	1641	1°47′22.68″W	40°20′32.94″N	50	7.54	1247
Spain, Cuenca (CU)	Arcas	ArCU	1099	2°04′04.38″W	39°54′27.00″N	50	10.94	735
Spain, Cuenca (CU)	Palancares	PaCU	1185	1°57′38.97″W	40°00′28.86″N	10	10.38	818
Morocco, Talassemtane (MO)	Talassemtane	TaMO	1710	5°08′26.63″W	35°08′30.33″N	10	9.86	1830
Spain, Sierra Mágina (MA)	Sierra Mágina	MA	1820	3°26′50.74″W	37°42′44.96″N	10	11.51	1128
Spain, Cazorla (CA)	Palancares	PaCA	1084	2°51′59.90″W	38°06′8.25″N	10	12.64	939
Spain, Cazorla (CA)	Navaciazo	NaCA	1400	2°51′43.84″W	37°53′45.75″N	10	10.45	1219
Spain, Cazorla (CA)	Yelmo	YeCA	1565	2°39′19.51″W	38°14′56.97″N	10	9.76	975

Elev.: elevation; *n*: number of samples in each population;

a Data collected from local forestry departments.
